# How Does the Canadian General Public Rate Moderate Alzheimer's Disease?

**DOI:** 10.4061/2011/682470

**Published:** 2011-12-20

**Authors:** Jean-Eric Tarride, Mark Oremus, Eleanor Pullenayegum, Natasha Clayton, Parminder Raina

**Affiliations:** ^1^Programs for Assessment of Technology in Health (PATH) Research Institute, St. Joseph's Healthcare Hamilton, 25 Main Street West, Suite 2000, Hamilton, ON, Canada L8P 1H1; ^2^Department of Clinical Epidemiology and Biostatistics, McMaster University, 1280 Main Street West, Hamilton, ON, Canada L8S 4L8; ^3^McMaster Evidence-Based Practice Centre, McMaster University, DTC Building, 3rd Floor, 50 Main Street East, Hamilton, ON, Canada L8N 1E9; ^4^Centre for Evaluation of Medicines, St. Joseph's Hospital, Martha Wing H321, 50 Charlton Avenue East, Hamilton, Canada L8N 4A6

## Abstract

*Objectives*. The objectives of this study were to elicit health utility scores for moderate Alzheimer's disease (AD) using members of the general public. *Methods*. Five-hundred Canadians were chosen randomly to participate in a telephone interview. The EQ-5D was administered to estimate the health utility score for respondents' current health status (i.e., no AD) and for a hypothetical moderate AD health state. Regression analyses were conducted to explain the perceived utility decrement associated with AD. *Results*. The mean age of the respondents was 51 years, 60% were female, and 42% knew someone with AD. Respondents' mean EQ-5D scores for their current health status and a hypothetical moderate AD were 0.873 (SD: 0.138) and 0.638 (SD: 0.194), respectively (*P* < 0.001). Age, gender, and education were significant factors explaining this decrement in utility. *Conclusion*. Members of the general public may serve as an alternative to patients and caregivers in the elicitation of health-related quality of life in AD.

## 1. Introduction

Alzheimer's disease (AD), the most common form of dementia, is the fifth leading cause of death in the United States (US) among persons aged 65 years or older [1]. Approximately 5.3 million Americans have AD, 5.1 million of whom are over the age of 65 years [2]. The financial burden of AD and other dementias is considerable, with an estimated annual total cost of $148 billion in the US in 2005 [3]. The annual cost per patient in the US was found to be three times higher for persons with AD and other dementias relative to persons without AD or other dementias (i.e., $33,007 relative to $10,603 in 2004) [3]. Other studies conducted in the US or elsewhere similarly concluded that AD costs were high and likely to rise over time due to the aging of the population [4–7]. 

Although several medications have been approved to treat AD (e.g., cholinesterase inhibitors, memantine), some jurisdictions (e.g., United Kingdom [8], Ontario, Canada [9]) limit reimbursement of these drugs (e.g., reimbursement in the United Kingdom is limited to persons with moderate AD only). The most prominent reasons for limiting reimbursement are grounded in the fact that the medications treat the symptoms of AD only (they are not a cure) [10] and cost between $2.90 and $6.80 per pill (US figures) [11]. In a cost-containment environment, economic evaluations are playing an increasingly important role in pricing and reimbursement decisions, [12], especially for relatively expensive medications that have modest efficacy.

In AD, cognitive decline continues when persons with the disease take cholinesterase inhibitors or memantine [10]. Consequently, an important outcome to consider when assessing these drugs in economic evaluations is health-related quality of life (HRQoL). In economic evaluations, HRQoL is usually expressed as part of a quality-adjusted life year (QALY). A QALY is a composite measure of outcome where utilities for health states (expressed on a 0-1 scale where 0 corresponds to death and 1 to perfect health) act as qualitative weights to combine the quantity and quality of life. Utilities can also be negative as some health states may be considered worse than death.

The primary means of eliciting utilities in AD is to administer a HRQoL questionnaire to patients or their caregivers (acting as patient proxies) and convert the resulting ratings into health utility scores using a scoring algorithm. At least two instruments, the quality of life-Alzheimer's disease (QoL-AD) [13] and dementia quality of life (DEMQOL) [14], have been developed to assess AD patients' HRQoL. Both instruments have been tested in caregiver proxies and baseline norms for a nondiseased population have been developed for the QoL-AD [15]. However, neither instrument has a scoring algorithm that can be used to convert HRQoL ratings into health utility scores. For these reasons, generic utility instruments such as the EuroQoL (EQ-5D) [16] and the Health Utility Index (HUI) [17] have also been used to elicit HRQoL for AD patients. These instruments have scoring algorithms that allow HRQoL ratings to be transformed into health utility scores. These generic measures are often preferred to disease-specific measures in economic evaluations for policy making to facilitate comparisons across diseases by using benchmarks (e.g., $50.000 per QALY gained). 

Several studies have shown that the EQ-5D [18–23] and the HUI [21, 22] may be used to reliably elicit utilities in mild-to-moderate AD patients. However, a number of studies [18, 20, 22, 24] also reported that some mild-to-moderate AD patients would rate their health as perfect (i.e., utility score of 1) which could be due to patients' lack of insight about their impairment [22, 25]. In addition, many AD patients do not consider themselves sick or suffering from AD [21]. To avoid the challenges of patient-measured HRQoL in AD, caregivers have been used as proxies to measure HRQoL for persons with AD. In such cases, caregivers complete instruments like the EQ-5D or the HUI on behalf of patients. Comparisons suggest caregivers' mean proxy health utility scores for patients are lower than patients' self-reported health utility scores on both the EQ-5D and HUI [19, 24, 26–28]. These differences may be due to the influence of caregiver-specific issues such as burden, which may be especially important when caring for moderate AD patients. One study of the EQ-5D and HUI reported that caregivers' proxy HRQoL ratings were associated with their own levels of burden, rather than patients' self-reported HRQoL ratings or patients' scores on the Mini-Mental State Examination [29] for cognitive impairment [24]. Conversely, the authors of the same study wrote that caregivers' proxy ratings on the EQ-5D or HUI are at least as reliable as patients' own ratings on these scales. 

The equivocal results in patients and caregivers suggest the need to search for other viable options that may be used to obtain estimates of AD patients' HRQoL and health utility. One option that has not been explored in the literature to date is to use the general public as a proxy to elicit HRQoL for AD patients. This alternative may be especially relevant for healthcare systems financed in whole or in part by public taxation (e.g., United Kingdom, Canada).

The first step in assessing the usefulness of the general public as a proxy is to determine whether the general public's HRQoL ratings would be sensitive to AD as a disease entity. That is, would the general public assign different HRQoL ratings to an AD disease state relative to a non-AD disease state? We had the opportunity to conduct a pilot study to address this question by using data collected for a project investigating Canadians' level of support for a tax increase to fund unrestricted access to AD medications [30]. In the taxation study, we conducted a telephone survey of 500 randomly-selected members of the Canadian general public who did not have AD. Part of the survey involved the administration of the EQ-5D. Participants were asked to complete the EQ-5D for their current health state and again for a health state of moderate AD. Although it is possible to derive utilities from the general public through direct measurements using either a standard gamble (SG) approach (iteratively find the probability p in which the individual is indifferent between living with an hypothetical “moderate AD” forever or taking a drug which can cure or kill him with the probability p) or a time trade-off (TTO) approach (hypothetically live with “moderate AD” forever or live shorter in a better state of health); these methods are more resource intensive and complicated to implement than the administration of a preexisting questionnaire (e.g., EQ-5D). For these reasons, respondents were asked in this pilot study to answer the EQ-5D under a hypothetical “moderate AD” health state.

Findings suggesting that the general public can act as a proxy rater for AD patients' HRQoL have important implications for research. Recruitment of a random sample of the general public is easier than recruiting a sample of AD patients or caregivers. Regional or national rosters of patients or caregivers do not exist, so research involving these persons often faces the challenge of recruiting subjects from a patchwork of advocacy organizations, support groups, and medical practices. If the general public can be used in place of patients or caregivers, then there would be a clear efficiency gain for researchers who wish to determine HRQoL and health utilities for AD patients. Such a gain would be important for analysts conducting economic evaluations in response to rapidly changing policy environments.

## 2. Materials and Methods

### 2.1. Study Design and Population

Using random digit dialing methodology, 500 adult members of the Canadian general public were chosen to participate in a 15–20-minute telephone interview. The sample was national in scope and stratified by five household income categories before tax (i.e., less than $20.000; $20.000 to less than $40.000; $40.000 to less than $60.000; $60.000 to less than $80.000; $80.000 or more). One hundred participants were included in each stratum. We stratified by income categories to avoid selection bias on the tax support questions [30].

For the current paper, survey responses were weighted [31] using income distributions from Statistics Canada to ensure that the results were representative of the Canadian population in terms of income distribution. For example, the 2006 Canadian Census indicated that 6.86% of the Canadian population had a total household income before tax of less than $20.000, [32] compared to 20% in our sample. In comparison, the higher household income category (≥$80.000 per year) was underrepresented in our sample (20% versus 39% in Canada). The composition of our sample was almost representative of the Canadian population for the three other household income categories (i.e., 18.02% of Canadians had a total household income before tax between $20.000 to $40.000; 19.34% of Canadians had a total household income before tax between $40.000 and $60.000; 17.10% of Canadians had a total household income before tax between $60.000 and $80.000). 

### 2.2. Data Collection

Data were collected using a structured computer-assisted telephone interview (CATI). During the interview, participants were asked to answer general questions about sociodemographics, knowledge of AD, health-related quality of life, and whether they knew someone with AD. Further details of the study methodology have been published elsewhere [30]. 

#### 2.2.1. Sociodemographics

Several variables were collected to control for sociodemographics differences between participants in terms of age, gender, income, education (high school or less; at least some technical or community college; at least some university), and employment (not working, working, or retired).

#### 2.2.2. Awareness of AD

AD awareness was measured in two ways. At study time, the Alzheimer's Disease Knowledge Test (ADKT) [33] was the only questionnaire to measure AD awareness. The ADKT includes five true or false statements (e.g., AD is a normal part of getting older, like gray hair and wrinkles: true or false?). Higher scores on this questionnaire represent a better knowledge of AD. In addition to the ADKT, participants were asked if they knew friends or relatives with AD.

#### 2.2.3. Health-Related Quality of Life

The EQ-5D was used to measure HRQoL. The EQ-5D is a validated, generic HRQoL questionnaire that measures health status in terms of five dimensions: mobility, self-care, usual activity, pain/discomfort, and anxiety/depression. The EQ-5D was developed to be self-administered, but it has been successfully used in telephone surveys of caregiver burden in AD [34] and chronic diseases in Canada [35]. Although single dimension scores are not available, the EQ-5D provides a weighted health utility score based on population values, ranging from 0 (death) to 1 (perfect). The United States population-based preference weights [36] were applied to the five EQ-5D questions to generate the EQ-5D health utility score. The EQ-5D questionnaire also includes a visual analogue scale (VAS) with anchors of 100 being the best imaginable state of health and 0 being the worst imaginable state of health. A score (i.e., 0–100%) from this self-rated “feeling thermometer” can be computed to indicate the subject's own assessment of their health state. 

The EQ-5D was administered twice in the study. First, participants were instructed at the beginning of the study to answer the EQ-5D thinking of their own health state at the time of the interview. After 10 to 15 minutes of questions to determine respondents' support for a tax increase to fund unrestricted access to AD medications, the following definition of moderate AD was verbally described to the participants: “*Alzheimer's disease is a progressive illness that causes memory loss and other cognitive deficits, advancing to major personality changes and eventual loss of control over bodily functions. In the moderate, mid-stage of Alzheimer's disease, mental abilities decline, personality changes, and physical problems develop so that the person becomes more and more dependent on caregivers*” [37]. After this description, participants were asked to answer the EQ-5D again, this time imagining they had moderate AD. We computed differences in health utility scores associated with the current health state and the hypothetical AD state. This allowed us to calculate the perceived decrement in utility due to AD (i.e., difference between utilities associated with one's own health state and the hypothetical AD state). 

### 2.3. Statistical Analyses

Continuous variables (e.g., age, income) were summarized using mean values and standard deviations. Discrete variables (e.g., gender, education) were summarized using percentages. The paired *t*-test was used to determine if differences in utility or VAS scores between the current health state and the hypothetical moderate AD state were significant. Statistical significance of differences between independent subgroups (e.g., comparing characteristics of those answering the EQ-5D assuming AD versus those who did not) was assessed using the *t*-test and Pearson's chi-squared statistical test for continuous and discrete variables, respectively. 

Regression analyses were conducted to gain a better understanding of the determinants of the EQ-5D utilities. Because utility data are often nonnormal, with a ceiling effect at 1, we used a model based on ordinary least squares (OLS) regression, coupled with bootstrap robust standard errors, to analyse the utility data [38]. When modelling the health utility score associated with the current health state of respondents, age, gender, education, and income categories were used as covariates. The score from the AD knowledge test and whether participants know someone with AD were also included as covariates because these factors may influence one's perception of a moderate AD health state. All descriptive statistics and regression outputs are presented in terms of weighted results. For comparison purposes, we also present the unweighted results for participants' sociodemographics characteristics. 

The study received ethics approval from the Hamilton Health Sciences/McMaster Faculty of Health Sciences Research Ethics Board (Reference no. 08–179).

## 3. Results

### 3.1. Study Participants

Table 1 presents participants' unweighted and weighted sample characteristics. Starting with the unweighted results, the mean age of the unweighted sample was 51.8 years (median age: 51.0 years). Most were female (61%) and working (56%), and 37% had at least some university education. Forty-two percent of the sample reported that they knew a family member or close friend with AD. As the sample was weighted to have more individuals in the highest income category and less in the lowest income category, higher education and employment rates were observed in our weighted sample. No differences were observed between the unweighted and the weighted sample in terms of average age (i.e., 51.8 versus 50.6, resp.) gender, or knowing someone with AD (Table 1). 

### 3.2. AD Awareness

**Figure 1:**
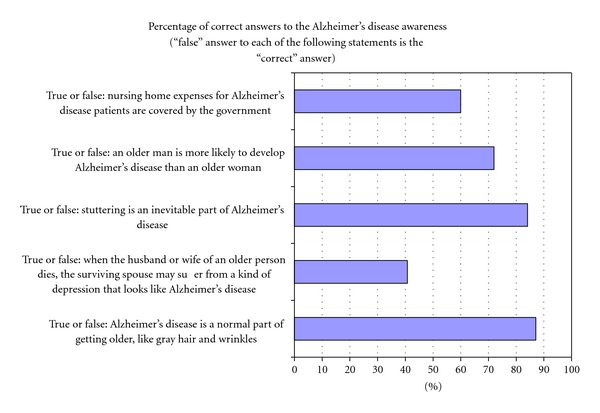
Alzheimer's disease knowledge test (ADKT) (weighted results).

The mean weighted ADKT score was 3.4 (SD: 1.0) out of 5 (higher scores indicate better knowledge of AD). This reflects the fact that the majority of the participants answered 4 out of 5 ADKT questions correctly. However, as shown in Figure 1, almost 60% of participants wrongly thought that depression following the death of a husband or wife was similar to AD. No statistical differences were found in the ADKT score between participants who knew someone with AD (i.e., 3.5) and those who did not (i.e., 3.4). The mean unweighted score was similar to the weighted score, that is, 3.4 (SD: 1.1).

### 3.3. EQ-5D Health Utility Score and Visual Analog Scale (VAS)

For the full sample (i.e., *n* = 500), the weighted mean EQ-5D utility score for participants' self-assessed current health state was 0.875 (SD: 0.137) and the mean VAS score was 80.79. Unweighted mean scores were 0.857 and 79.16, respectively. 

While 99% of all participants were able to rate their current health state on the EQ-5D, approximately 14% of participants were unable to answer all five EQ-5D questions for a hypothetical, moderate AD health state. In total, health utility scores for a moderate AD health state were calculated for 431 participants (i.e., 86%). When participants with complete (*n* = 431) or incomplete EQ-5D (*n* = 69) values were compared, only age was found to be significantly different. Participants with missing data were older than participants with nonmissing data (56.0 years of age versus 49.7 years of age, *P* = 0.0011). 

**Figure 2:**
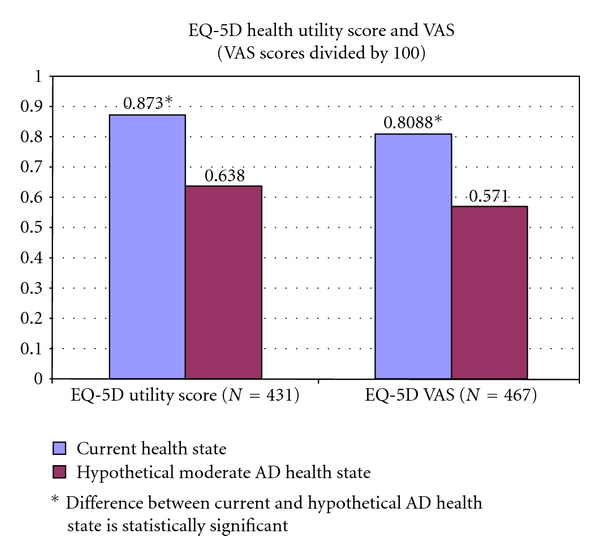
Current health state versus hypothetical moderate AD state (weighted results).

Figure 2 presents the mean weighted EQ-5D health utility score and VAS scores for the current health state and the hypothetical moderate AD health state for the 431 and 467 participants with complete EQ-5D utility score and VAS data, respectively. The weighted EQ-5D health utility score for current health state was 0.873 (SD: 0.138) and 0.638 (SD: 0.194) for a hypothetical moderate AD health state (versus unweighted means of 0.856 and 0.639, resp.). The difference between the current health state and the hypothetical health state were statistically significant (*P* < 0.0001) in both the weighted and unweighted cases. The utility decrement associated with moderate AD was 0.235 for the weighted results and 0.217 for the unweighted results.

Four hundred and sixty seven participants (93% of all participants) answered the VAS for the current health state and the hypothetical health state. On a scale from 0 to 100, the weighted VAS scores decreased significantly from 80.88 (current health state) to 57.10 (moderate AD health state) (*P* value < 0.0001). The unweighted VAS scores were 79.38 and 57.70, respectively (*P* value < 0.0001). As shown in Table 2, participants perceived they would have reductions in mobility, self-care, and usual activities, as well as more depression, when in a state of moderate AD. 

### 3.4. Regression Analyses

Table 3 presents the results of the two regression analyses used to identify the determinants of the utility scores associated with the current health state and the hypothetical moderate AD health state. In explaining the current health state, age and income categories were significant variables, while education and gender were not. Utility decreased with age and increased with income. When analyzing the utility decrement associated with moderate AD (modeled as the difference between utility associated with the current health state and the hypothetical AD health state), age, gender, and a university background were significant variables. As age increases, the difference in health utility score decreases between the current health state and the hypothetical health state. Women compared to men, and more educated participants, were more likely to perceive a higher decrement in utility due to AD. Income, knowledge of AD, or knowing someone with AD had no impact on utility decrement. Results were similar for the unweighted regressions. 

## 4. Discussion

This pilot study demonstrates for the first time that the general public's HRQoL ratings and health utility scores are sensitive to AD as a disease entity. Severe reductions in mobility, self-care, and usual activities, and increased depression, were perceived when members of the general public answered the EQ-5D under the assumption that they had moderate AD. Mean utilities were also lower for moderate AD relative to the current health state. Interestingly, greater knowledge of AD and knowing someone with AD did not influence the perception of AD, while age, gender, and having a university diploma were significant variables. This may suggest that sociodemographics factors may be more important than awareness of disease when the general public is used as a proxy for assessing AD patients' HRQoL. 

This pilot study has several strengths. The participants were recruited from a pan-Canadian sampling frame using a random sampling methodology, thus eliminating selection biases associated with region of residence or location of recruitment. 

Data from 500 individuals were collected with a standardized interview that was conducted by trained interviewers using CATI software to lessen potential information bias. Of course, the limitations inherent in telephone surveys, for example, a sample frame containing only persons who have a home telephone number and nonresponse bias, also apply to our study. Our participants may be different from persons who were unreachable by telephone or unwilling to participate.

Another issue regarding our study is the composition of the sample, which may not be entirely representative of the Canadian population. Since the sample was stratified by income categories to minimize bias in determining levels of support for a tax increase [30], we had to weight the responses using Canadian income distributions. Minimal differences were observed between the weighted and unweighted results, suggesting that the stratification of our sample was unlikely to have an impact on the results.

Our study was a pilot project using data collected to answer an unrelated research question about support for a tax increase to fund unrestricted access to AD medications [30]. Thus, we were limited to assessing HRQoL using the EQ-5D, which was the only HRQoL instrument employed in the tax support study. We recognize the limitations of the EQ-5D in assessing HRQoL from AD patients and caregiver proxies [21, 22, 24]. However, one should note that the EQ-5D has not been used with general public proxies to estimate AD patients' HRQoL. Our results suggest the EQ-5D may be useful with general public proxies, although work is needed to assess its test-retest reliability in the general public. Due to our study design (e.g., telephone survey) and time constraints, we did not use the standard gamble or time trade-off techniques and it is currently unknown to which extent these different methods would differ when using the general public as a proxy for utility assessment in AD. However, all methods (e.g., standard gamble or EQ-5D) have in common that they ask respondents to imagine they had moderate AD. Future research should compare utility data derived from patients, caregivers, and general public. 

In the tax support study, we collected data for proxy ratings of moderate AD only. Future research needs to assess the general public's proxy ratings for mild and severe AD. As well, we used the ADKT to examine participants' level of knowledge regarding AD. We realize the ADKT is dated, but at the time of data collection (i.e., 2008), the ADKT was the only test available to gauge what people knew about AD. In the future, researchers surveying the general public may wish to incorporate a newer instrument that became available in 2009, that is, the Alzheimer's Disease Knowledge Scale [39]. The general public's ratings on the EQ-5D may have been influenced by the specific scenario we used to describe moderate AD. Future research should test for “scenario effects” by utilizing more than one description of each AD state and randomizing participants to receive different sets of descriptions. 

Our pilot results cannot easily be compared to previous studies because our work is the first attempt to measure utilities for a moderate AD health state using the general public as a proxy. We did find the health utility index score associated with moderate AD in our study (i.e., 0.65) to be similar to previous Canadian (i.e., 0.62) [22] and US (0.65) [24] findings where caregivers were used as proxies for mild-to-moderate AD patients. Despite some of the aforementioned issues with caregiver proxy ratings, the caregiver ratings were very close to the general public ratings. However, more research needs to be undertaken to compare AD patients' ratings with general public and caregiver proxy ratings to assess potential validity issues, for example, the possibility of inverse correlations between patient ratings and general public proxy ratings. Nonetheless, our results suggest that in the absence of caregiver or patient data, the general public could be used as proxy to elicit utility data that could be used in decision analytic models comparing treatment options for AD in terms of incremental cost per QALY gained. 

## 5. Conclusions

This study is the first research project using the general public to elicit HRQoL and health utility scores for moderate AD. When utility is captured by the EQ-5D, the general public believes that their utility would decrease by 0.235 if they had moderate AD. This result suggests the general public is sensitive to quality decrements in AD; members of the general public may serve as an alternative to patients and caregivers in the elicitation of HRQoL and heath utilities in AD which could be used for economic evaluations. This is important from a methodological perspective because there are fewer barriers to conducting a telephone survey of a random sample of the general public relative to recruiting a sample of caregivers or AD sufferers. 

## Figures and Tables

**Table 1:** Characteristics of participants (unweighted and weighted samples).

Sample characteristics	Unweighted sample		Weighted sample
Gender	*n*	%	%
Female	305	61	60
Male	195	39	40
Annual household income			
<$20.000	100	20	7
$20.000 to <$40.000	100	20	18
$40,000 to <$60.000	100	20	19
$60.000 to <$80.000	100	20	17
≥$80.000	100	20	39
Education			
High school or less	169	34	26
At least some technical or community college	143	29	30
At least some university	184	37	44
Missing	4	<1	<1
Employment status			
Not working (unemployed, student, homemaker)	66	13	10
Retired	142	28	24
Employed (full or part time)	281	56	64
Missing	11	2	2
Participant knows a family member or close friend with			
Alzheimer's disease			
Yes	211	42	42
No	286	57	57
Missing	3	1	1

**Table 2:** Domains of EQ-5D: current and hypothetical moderate AD health state (weighted results).

Domains/questions	Current health state (*N* = 431)	Assuming AD (*N* = 431)
*Mobility*		
I have no problems in walking about	85.92%	42.80%
I have some problems in walking about	14.08%	55.97%
I am confined to bed	0%	1.23%
*Self-care*		
I have no problems with self-care	96.69%	27.12%
I have some problems washing or dressing myself	3.31%	71.13%
I am unable to wash or dress myself	0.0%	1.75%
*Usual activities *		
I have no problems with performing my usual activities	82.67%	16.68%
I have some problems with performing my usual activities	15.67%	72.84%
I am unable to perform my usual activities	1.66%	10.48%
*Pain/discomfort*		
I have no pain or discomfort	52.77%	42.14%
I have moderate pain or discomfort	45.20%	55.78%
I have extreme pain or discomfort	2.02%	2.09%
*Anxiety/depression*		
I am not anxious or depressed	75.61%	18.73%
I am moderately anxious or depressed	22.78%	56.96%
I am extremely anxious or depressed	1.61%	24.31%

**Table 3:** Regression analyses: EQ-5D utility score and perceived utility decrement associated with a hypothetical moderate AD health state (weighted results).*

Variable	Category	EQ-5D health utility score self-assessed current health state		Utility decrement associated with moderate AD health state	
		Coefficient	Bootstrap SE*	Coefficient	Bootstrap SE*
Intercept	—	**0.8308**	**0.0349**	0.2650	0.0580
Age	Years	**−0.0014**	**0.0004**	**−0.0035**	**0.0007**
Gender	Female versus male	0.0170	0.0131	**0.0480**	**0.0218**
Some college	Versus high school or less	−0.0030	0.0201	0.0441	0.0297
Some university	Versus high school or less	0.0329	0.0194	**0.0784**	**0.0256**
Income: $20 to 40 K	Versus less than $20 K	**0.1019**	**0.0256**	0.0254	0.0349
Income: $40 to 60 K	Versus less than $20 K	**0.0841**	**0.0258**	−0.0062	0.0361
Income: $60 to 80 K	Versus less than $20 K	**0.0999**	**0.0267**	0.0504	0.0368
Income: $80 K and +	Versus less than $20 K	**0.1065**	**0.0279**	0.0479	0.0333
Know someone with AD	Versus not knowing	**—**	**—**	0.0032	0.0215
ADKT score	—	**—**	**—**	0.0107	0.0099
Multiple *R*-squared		0.1028		0.1415	
Adjusted *R*-squared		0.0877		0.1208	

*Bold indicates statistical significance (i.e. *P* < 0.05).
